# Antioxidant Activity of a Mediterranean Food Product: “Fig Syrup”

**DOI:** 10.3390/nu3030317

**Published:** 2011-02-28

**Authors:** Francesco Puoci, Francesca Iemma, Umile G. Spizzirri, Donatella Restuccia, Vincenzo Pezzi, Rosa Sirianni, Lillo Manganaro, Manuela Curcio, Ortensia I. Parisi, Giuseppe Cirillo, Nevio Picci

**Affiliations:** 1 Pharmaceutical Sciences Department, University of Calabria, Edificio Polifunzionale, Arcavacata di Rende (CS) 87036, Italy; Email: francesca.iemma@unical.it (F.I.); g.spirizzi@unical.it (U.G.S.); donatella.restuccia@unical.it (D.R.); drlillo.manganaro@gmail.com (L.M.); manuela.curcio@unical.it (M.C.); ortensiailaria.parisi@unical.it (O.I.P.); giuseppe.cirillo@unical.it (G.C.); nevio.picci@unical.it (N.P.); 2 Department of Pharmaco-Biology, University of Calabria, Edificio Polifunzionale, Arcavacata di Rende (CS) 87036, Italy; Email: v.pezzi@unical.it (V.P.); rsirianni@unical.it (R.S.)

**Keywords:** antioxidants, nutraceutical supplement, fruits/vegetables analysis, enzyme inhibition, polyphenols, fig syrup

## Abstract

In this work, the efficacy of fig syrup, a Mediterranean fig derivative, as a nutraceutical supplement, was demonstrated. Fig syrup is a fruit concentrate used as a common ingredient in the preparation of typical foods, and particularly in cakes. *In vitro* assays were performed to determine the amount of nutraceutical ingredients, such as phenolic compounds (3.92 mg equivalent of gallic acid per g) and flavonoids (0.35 mg equivalent of catechin per g), while HPLC analyses provided specific information about the composition of antioxidants in the syrup. Furthermore, total antioxidant activity, scavenging properties against DPPH and peroxyl radicals, and the anticholinesterase activity, clearly showed the efficacy of the syrup in preventing damage induced by free radicals and, thus, the applicability of this food derivative as a nutraceutical supplement.

## 1. Introduction

The Mediterranean diet has been reported to promote health and quality of life in those who adhere to it, specifically by preventing pathophysiological conditions related to coronary heart disease and cancer [1]. The high consumption of natural antioxidants, achieved by consuming salads, vegetables, fruits, and their derived products, is generally considered to be a major beneficial contributor to the Mediterranean diet [2]. Various diseases including arthritis, cirrhosis, emphysema, atherosclerosis and cancer are believed to be correlated with the oxidative damage induced by these free radicals [3]. 

Therefore, oxidative damage plays a significant pathological role in human disease [4,5]. 

Antioxidants may offer resistance to oxidative stress by scavenging free radicals, inhibiting lipid peroxidation, *etc.*, and thus prevent the onset of deadly diseases. Apart from the antioxidants synthesized naturally, the body requires a supplement of dietary antioxidants, which can be obtained only by the consumption of an antioxidant-rich diet [6].

Thus, the ingestion of antioxidative supplements or foods containing antioxidants is now widely considered an effective strategy to reduce oxidative damage and exert a beneficial effect on human health [7,8,9]. 

Vegetables are good sources of natural antioxidants, such as carotenoids, vitamins, flavonoids, and other phenolics compounds [10,11,12]. Many studies show that daily consumption of fruits and vegetables is associated with reduced risks for chronic degenerative diseases, because they contribute polyphenols as well as antioxidant vitamins to the diet [13]. Attention has therefore been focused in recent years on antioxidant products from natural sources, which are very attractive to the food industry, prompting their use as replacements for synthetic antioxidants and also as nutraceuticals, playing a role in preventing many diseases [14].

Fig is one of the most abundant fruits in the Mediterranean diet [15] and an artisan derivative of this fruit is “Fig Syrup”, a typical Calabrian food product made by boiling and concentrating fresh figs (generally the cultivar is *Ficus carica* also known as “Fico Dottato Calabrese”) in water, without adding any other ingredient. The obtained product is a dense syrup characterized by its brown color, sweet taste and smell. It is widely found in the Mediterranean region, and in Italy the production area matches the old “Kingdom of the Two Sicilies”. For a long time in Calabria, it has been a common ingredient for the preparation of typical foods and for cakes in particular. 

The aim of the present study is to characterize the Calabrian fig syrup in terms of antioxidant properties, in order to prove the applicability of this food product as a nutraceutical supplement as reported for other well known natural syrups such as maple syrup [14]. The analysis of phenolic compounds from fresh fig is reported in literature [15], whereas none have investigated the antioxidant and antiradical activities of this fig derivative. The advantage of using fig syrup consists of the ease of handling and the possibility to employ a fruit concentrate characterized by a very high amount of nutraceutical compounds.

Syrup was characterized by High-performance liquid chromatography (HPLC)–diode array detection (DAD)–mass spectrometry (MS) analyses, to determine the composition of the nutraceutical formulation. A 2,2’-diphenyl-1-picrylhydrazyl radical (DPPH) assay was performed to evaluate the scavenger activity; Folin-Ciocalteau and AlCl_3_ tests were employed to determine the total phenolic content and flavonoids, respectively; molybdate reactive was employed to evaluate the total antioxidant capacity and finally a β-Carotene-linoleic Acid assay was performed to evaluate the capacity of inhibiting the lipid peroxidation. Finally, a specific assay was performed to evaluate the anticholinesterase activity of the fruit derivative.

## 2. Experimental Section

### 2.1. Sample

Fig syrup was obtained from a local artisan producer in Cosenza (Calabria) and was diluted in water (1/50, w/w) and filtered before use.

### 2.2. Chemicals

2,2’-Diphenyl-1-picrylhydrazyl radical (DPPH), Folin-Ciocalteu reagent, sodium carbonate, sulfuric acid (96%, w/w), trisodium phosphate, ammonium molybdate, β-carotene, linoleic acid, Tween 20, sodium nitrite, aluminum chloride, sodium hydroxide, gallic acid, catechin, epicatechin, rutin, chlorogenic acid, syringic acid, *Torpedo californica* (electric eel) acetylcholinesterase (Type-VI-S, EC 3.1.1.7 (AChE)), acetylthiocholine iodide (ATCI), physostigmine and 5,5’-dithiobis (2-nitrobenzoic-acid) (DTNB), were obtained from Sigma-Aldrich (Sigma Chemical Co., St Louis, MO, USA).

Ethanol and chloroform were HPLC-grade and provided by Fluka Chemika-Biochemika (Buchs, Switzerland).

### 2.3. Instrumentation

UV-Vis absorption spectra were obtained with a Jasco V-530 UV/Vis spectrometer.

High-performance liquid chromatography (HPLC)–diode array detection (DAD)–mass spectrometry (MS) analyses were performed using an Agilent 1100 Series LC/MSD system with a diode array detector (DAD) coupled to a mass spectrometer (quadrupole analyzer) equipped with an electrospray ionization interface (ESI, Agilent). Chromatographic separation was carried out using a 150 × 4.6 mm i.d., 5 μm SS Wakosil C18 with a 4 × 3 mm i.d. Phenomenex C18 guard cartridge, both thermostated at 32 °C.

The elution solvents used were A (aqueous 0.01 M phosphoric acid) and B (100% methanol). The samples were eluted according to the following gradient: 5% B as initial condition; 50% B for 10 min; 70% B for 5 min, 80% B for 5 min and finally 100% in B for 5 min. The chromatographic data on the peaks were integrated up to 25 min. The flow-rate was 0.3 mL/min. The sample injection volume was 20 μL. The diode array scanned three discrete channels at 280, 365, and 520 nm.

MS parameters were as follows: capillary voltage, 4000 V; fragmentor, 160 V; drying gas temperature, 350 °C; gas flow (N_2_), 10 L/min; nebulizer pressure, 50 psig. The instrument was operated in positive ion mode scanning from m/z 100 to 800 at a scan rate of 1.43 s/cycle.

Once components had been putatively identified by their relative retention, their PDA spectra and their mass spectral properties, the amount of specific components was estimated using the peak area calculated under the specific *m/z* value for each molecular species, as defined by the software associated with the mass spectrometer. This method gives a reasonable and internally comparable estimate of content even when peaks were not completely separated.

### 2.4. Extraction Procedure for LC/MS Analyses

The samples were prepared according to the literature [16]: 10 g of dried syrup was extracted with methanol containing 1% 2,6-ditert-butyl-4-methylphenol (dibutylhydroxytoluene, BHT), using an ultrasonic bath.

Samples were extracted with 10 mL of solvent for 1 h, 10 mL for 30 min, and finally 5 mL for 30 min. The three extraction fractions were combined into a final volume of 25 mL. Solutions to be analyzed by HPLC were filtered through a membrane filter (0.45 μm pore size) prior to injection. BHT was added to the samples to prevent oxidation during the extraction.

### 2.5. Determination of Scavenging Effect on DPPH Radicals

In order to evaluate the free radical scavenging properties of fig syrup, its reactivity towards the stable free radical DPPH was evaluated [17]. A solution of DPPH in ethanol (200 μM) was prepared freshly. A 5 mL aliquot of this solution was mixed with 5 mL of five different syrup solutions to raise the final concentrations to 0.2, 0.5, 1.0, 2.0, 4.0 mg/mL, respectively. The solutions in the test tubes were shaken well, incubated in the dark for 30 min at room temperature, and the absorbance of the remaining DPPH was determined colorimetrically at 517 nm. The scavenging activity of the syrup was measured as the decrease in absorbance of the DPPH and it was expressed as percent inhibition of DPPH radicals calculated according to the following Equation 1:


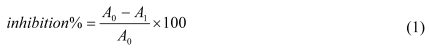


where *A*_0_ is the absorbance of a standard that was prepared in the same conditions, but without syrup, and *A*_1_ is the absorbance of syrup samples. Ascorbic acid was used as positive control.

### 2.6. Determination of Total Phenolic Content

Amount of total phenolic compounds was determined using Folin-Ciocalteu reagent procedure, according to the literature with some modifications [18]. A 6 mL aliquot of five different syrups was mixed thoroughly with 1 mL of Folin-Ciocalteu reagent in a volumetric flask. After 3 min, 3 mL of Na_2_CO_3_ (7.5%) was added, and then the mixture was allowed to stand for 2 h with intermittent shaking. The final concentrations of fig syrup in the samples were 0.2, 0.5, 1.0, 2.0, 4.0 mg/mL, respectively. The absorbance of the solutions was measured at 760 nm to record a calibration curve and the correlation coefficient (*R*^2^), slope and intercept of the regression equation obtained were calculated by the method of least square.

The amount of total phenolic compounds in the syrup was expressed as mean (milligrams of gallic acid equivalents per gram of syrup) ± SD for five replications, by using the equation obtained from the calibration curve of the antioxidant. This was recorded by employing five different gallic acid standard solutions in the same conditions reported for the syrup. The final concentrations of gallic acid in the test tubes were 8.0, 16.0, 24.0, 32.0, and 40.0 μM, respectively. 

### 2.7. Determination of Flavonoids

A slightly modified version of the spectrophotometric method was used to determine the flavonoid contents of samples [19]. Briefly, in five test tubes 0.25 mL of five different fig syrup solutions were mixed with 1.25 mL of distilled water followed by addition of 75 μL of a 5% NaNO_2_ solution. After 6 min, 150 μL of a 10% AlCl_3_**·**6H_2_O solution were added and allowed to stand for another 5 min before 0.5 mL of 1 M NaOH was added. The mixture was brought to 2.5 mL with distilled water and mixed well. The final concentrations of fig syrup in the samples were 0.2, 0.5, 1.0, 2.0, 4.0 mg/mL, respectively. The absorbance was measured immediately against the blank at 510 nm to record the calibration curve and the correlation coefficient (*R*^2^), slope and intercept of the regression equation obtained were calculated by the method of least square.

The amount of total flavonoids in the syrup was expressed as mean (milligrams of catechin equivalents per gram of syrup) ± SD for five replications, by using the equation obtained from the calibration curve of the antioxidant. This was recorded by employing five different catechin standard solutions with the same procedure. The final concentrations of catechin in the test tubes were 10, 25, 50, 75, 100 μM, respectively.

### 2.8. Determination of Total Antioxidant Activity

The total antioxidant activity of fig syrup was evaluated according to the method reported in literature [20]. Briefly, in five test tubes, 0.3 mL of five different fig syrup solutions were mixed with 1.2 mL of reagent solution (0.6 M sulphuric acid, 28 mM sodium phosphate and 4 M ammonium molybdate) to rise the final concentrations of 0.2, 0.5, 1.0, 2.0, 4.0 mg/mL, respectively. Then the reaction mixture was incubated at 95 °C for 150 min and, after cooling to room temperature, the absorbance of the mixture was measured at 695 nm to record the calibration curve. The correlation coefficient (*R*^2^), slope and intercept of the regression equation obtained were calculated by the method of least square.

The total antioxidant activity of the syrup was expressed as mean (micrograms of gallic acid equivalents per gram of syrup) ± SD for five replications, by using the equation obtained from the calibration curve of the antioxidant. This one was recorded by employing five different gallic acid standard solutions with the same procedure. The final concentrations of gallic acid in the test tubes were 8.0, 16.0, 24.0, 32.0, and 40.0 μM, respectively. 

### 2.9. β-Carotene-linoleic Acid Assay

The antioxidant properties of fig syrup were also evaluated through measurement of percent inhibition of peroxidation in linoleic acid system by using the β-carotene bleaching test [21]. Briefly, 1 mL of β-carotene solution (0.2 mg/mL in chloroform) was added to 0.02 mL of linoleic acid and 0.2 mL of Tween 20. The mixture was then evaporated at 40 °C for 10 min in a rotary evaporator to remove chloroform. After evaporation, the mixture was immediately diluted with 100 mL of distilled water. The water was added slowly to the mixture and agitated vigorously to form an emulsion. The emulsion (5 mL) was transferred to seven different test tubes and 0.5 mL of fig syrup solutions were added to rise the final concentrations of 0.2, 0.5, 1.0, 2.0, 4.0, 6.0, 8.0 mg/mL, respectively. The tubes were then gently shaken and placed in a water bath at 45 °C for 60 min. The absorbance of the filtered samples and control was measured at 470 nm against a blank, consisting of an emulsion without β-carotene. The measurement was carried out at the initial time (*t* = 0) and successively at 60 min. 

The antioxidant activity (*A_ox_A*) was measured in terms of successful bleaching of β-carotene using the following Equation 2:


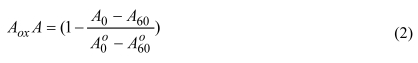


where *A*_0_ and *A*_0_^o^ are the absorbance values measured at the initial incubation time for samples and control, respectively, while *A*_60_ and *A*_60_^o^ are the absorbance values measured in the samples and in control, respectively, at *t* = 60 min. Propyl gallate was used as positive control.

### 2.10. Cholinesterase Inhibitory Assay

Inhibition of AChE were assessed by a modified colorimetric Ellman’s method [22], which is based on the reaction of released thiocoline to give a colored product with chromogenic reagent. Torpedo californica (electric eel) AChE (Type-VI-S, EC 3.1.1.7, Sigma) was used, while acetylthiocholine iodide (ATCI) was used as the substrate of the reaction. 40 μL of AChE (0.36 U/mL in buffer pH 8) and 20 μL of syrup at different concentrations were added to 2 mL of buffer pH 8 (0.1 mM) to raise the final concentrations of 1.0, 2.5, 5.0, 7.5, 10.0 mg/mL and pre-incubated in an ice bath at 4 °C for 30 min. Duplicate tubes were also treated this way with 20 μL of physostigmine (0.1 mM) to allow interference of the test substances in the assay to be assessed, and to control any hydrolysis of acetylcholine not due to enzyme activity. The reaction was started by adding DTNB solution (20 μL of 0.05 mM in buffer pH 7) and ATCI (20 μL 0.018 mM in buffer pH 7) and tubes were allowed to stand in a water bath for 20 min at 37 °C. The reaction was halted by placing the assay solution tubes in an ice bath and adding physostigmine (20 μL 0.018 mM in buffer pH 7). The hydrolysis of acetylthiocholine was monitored by the formation of the yellow 5-thio-2-nitrobenzoate, immediately recorded on a spectrophotometer at 405 nm and the percentage inhibition (%) was calculated by the Equation 3: 


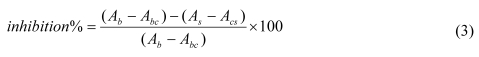


where *A*_b_ and *A*_bc_ are the absorbances of blank, and blank positive control, respectively, while *A*_s_ and *A*_cs_ are the absorbance of sample and sample positive control, respectively.

In this assay, physostigmine acted as positive control.

### 2.11. Statistical Analyses

The results are presented as the average of five experiments and standard deviation (±SD). Data were analyzed using one-way analysis of variance (ANOVA), and differences were considered significant at *P* < 0.05. The inhibitory concentrations 50% (IC_50_) were obtained by interpolation from linear regression analyses.

## 3. Results

### 3.1. Separation and Identification

A deep investigation of the phenolic content of the syrup was performed by HPLC-DAD-MS analyses. For this purpose, the food supplement was subjected to extraction procedure using methanol as a solvent [16]. The resulting extract, was then analyzed by the HPLC protocol and the following phenolic compounds were identified: gallic acid, chlorogenic acid (5-*O*-caffeoylquinic acid), epicatechin, catechin, syringic acid, and rutin (quercetin-3-*O*-rutinoside). The quantification of the compounds is reported in Table 1. Rutin was present in the highest concentrations among all the phenolics analyzed, while in the group of phenolic acids, the highest amounts were exhibited in the case of chlorogenic acid, followed by gallic acid, with trace amounts of syringic acid. Furthermore, although data demonstrated that fig syrup does not belong to food supplements rich in catechins (epicatechin and catechin), Table 1 shows that the amount of catechin is the second most abundant and higher than the total amount of the three phenolic acids.

**Table 1:** Content (mg per g syrup) of various phenolics in fig syrup (means ± SD are presented).

Compound	Amount
Gallic acid	0.021 ± 0.003
Chlorogenic acid	0.10 ± 0.04
Syringic acid	0.008 ±0.002
Epicatechin	0.007 ± 0.001
Catechin	0.34 ± 0.02
Rutin	1.9 ± 0.08

### 3.2. Determination of Scavenging Effect on DPPH Radicals

The scavenging of hydrogen radicals is one of the important mechanisms of antioxidation. In this study, DPPH was used to determine the free radical scavenging activity of fig syrup [23]. 

**Figure 1:**
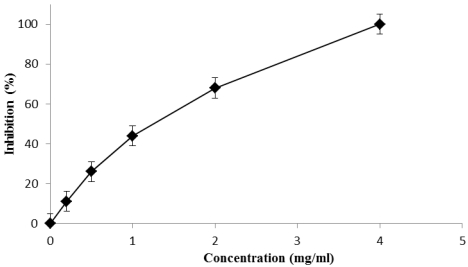
DPPH radical scavenging effect on different concentrations of fig syrup after 30 min incubation.

Fig syrup scavenger ability was evaluated in terms of DPPH reduction at different syrup concentrations, and data are expressed as inhibition (percent) as reported in Figure 1. 

The results show that the scavenging activity of the syrup is dose dependent and the half-inhibition concentration, IC_50_, which is the efficient concentration required to reduce initial DPPH concentration by 50%, is also calculated. This value was found to be 1.06 mg/mL for the syrup and 2.03 µg/mL for the positive control.

### 3.3. Total Phenolic and Total Flavonoid Content

The content of total phenolics was determined by employing Folin-Ciocalteau [24] reagent, and was expressed as mg of gallic acid per g of syrup.

By performing the test on different syrup concentrations, a calibration curve was recorded, and the correlation coefficient (*R*^2^ = 0.995), slope and intercept of the regression equation obtained were calculated by the method of least square. By comparing the data with the gallic acid calibration curve, a value of 3.92 mg of gallic acid equivalents per g of extract was determined.

The flavonoids content was determined by AlCl_3_ assay and expressed as mg of cathechin per g of syrup.

By performing the test on different syrup concentrations, a calibration curve was recorded, and the correlation coefficient (*R*^2^ = 0.992), slope and intercept of the regression equation obtained were calculated by the method of least square. By comparing the data with the catechin calibration curve, the amount of catechin equivalent (0.35 mg per g of extract) was determined. 

### 3.4. Determination of Total Antioxidant Activity

The assay is based on the reduction of Mo(VI) to Mo(V) by antioxidant compounds and subsequent formation of a green phosphate/Mo(V) complex at acid pH [20].

Total antioxidant activity of the syrup was expressed as mg equivalent of gallic acid per g of syrup. By performing the test on different syrup concentrations, a calibration curve was recorded, and the correlation coefficient (*R*^2^ = 0.993), slope and intercept of the regression equation obtained were calculated by the method of least square. By comparing the data with the gallic acid calibration curve, the amount of gallic acid equivalent (5.76 mg per g of extract) was determined.

### 3.5. β-Carotene-linoleic Acid Assay

**Figure 2:**
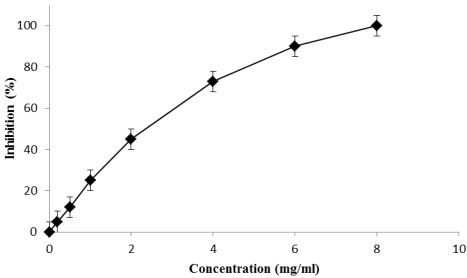
Lipid peroxidation inhibition capacity of fig syrup after 60 min incubation at 60 °C.

This test is a measure of the sample ability to inhibit lipid peroxidation. The inhibition percentages of lipid peroxidation by fig syrup were evaluated at different concentrations as shown in Figure 2. The results showed a correlation between the inhibition (%) and the concentration (mg/mL). The IC_50_ value was found to be 2.04 mg/mL for the syrup and 1.21 µg/mL for the positive control.

### 3.6. Cholinesterase Inhibitory Profile

Fig syrup was tested as AChE inhibiting agent and good activity was found with a dose dependence. The IC_50_ value was found to be 5.02 mg/mL, confirming that this fruit derivative is of particular relevance in nutraceutical supplementation. The IC_50_ value for the positive control physostigmine is 0.12 µg/mL.

## 4. Discussion

### 4.1. Separation and Identification

Fruits and vegetables containing high concentrations of chemicals have attracted considerable interest in recent years due to their potential health-promoting effects. These effects are at least partly due to their antioxidant capacity [25]. The total amount of electron-donating antioxidants in the diet is derived from combinations of individual antioxidants that occur naturally in food [26]. For these reasons, HPLC-DAD-MS analyses were performed in order to determine the chemical composition of fig syrup in terms of polyphenols and flavonoids content. The data reported in Table 1, shows that fig syrup is characterized by high levels of antioxidant compounds. 

The phenolics analyzed in our experiment were gallic acid, catechin, epicatechin, chlorogenic acid, syringic acid and rutin. 

Rutin (a quercetin glycoside) is the polyphenol with the highest concentrations in the sample. The presence of this compound is of great importance in a food supplement because quercetin is a flavonoid with high antioxidant and biological properties and rutin is much more easily taken up by the human body in the form of glycosides, which are afterwards transformed into active quercetin [27].

Chlorogenic acid is a phenolic acid, which is very common in different parts of plants and fruit and it was shown [28] that catechin and chlorogenic acid were equally effective as apple extracts in preventing oxidative injury to human gastric epithelial cells *in vitro*. However, chlorogenic acid is poorly absorbed in the human body and is metabolized by colonic microflora [29]. 

Gallic acid and its glycosides are characteristic of some berry crops, like currant, raspberry or strawberry [30]. It is extremely well absorbed into the human body, compared with other polyphenols [31] and shows high antioxidant activities as well as a positive effect under *in vitro* conditions against cancer cells [32]. 

Syringic acid is present in very low amount in the sample and it is reported to act as an antioxidant *in vitro* [33]. 

Epicatechin and catechin belong to the group of catechins which are reported to possess numerous biological activities, of which preventive effects against cancer are the most notable [34].

### 4.2. Antioxidant and Enzymatic Properties

After the chemical characterization, the scavenging ability of fig syrup towards DPPH radical was verified. 

The DPPH radical is a stable organic free radical with an absorption maximum band around 515–528 nm and, thus, is a useful reagent for evaluation of the antioxidant activity of compounds. In the DPPH test, the antioxidants reduce the DPPH radical to a yellow compound, diphenylpicrylhydrazine, and the extent of the reaction depends on the hydrogen-donating ability of the antioxidants. It has been documented that cysteine, glutathione, ascorbic acid, tocopherols, and polyhydroxy aromatic compounds (e.g., caffeic acid, hydroquinone, catechin, gallic acid) reduce and decolorize the 1,1-diphenyl-2-picrylhydrazyl radical (DPPH) by their hydrogen-donating capabilities. The obtained low IC_50_ value shows the good scavenging activity of the syrup. 

The Folin–Ciocalteu phenol reagent is used to obtain a crude estimate of the amount of total phenolic compounds present in the sample. Phenolic compounds undergo a complex redox reaction with phosphotungstic and phosphomolybdic acids present in the Folin–Ciocalteu reactant. The color development is due to the transfer of electrons at basic pH to reduce the phosphomolybdic/phosphotungstic acid complexes to form chromogens in which the metals have lower valence [24]. It has frequently been reported that both phenolic compounds and flavonoids are closely associated with antioxidant activity [18]. Thus, AlCl_3_ assay was employed to have a direct determination of the total flavonoid content of the syrup.

Then, the total antioxidant activity of syrup was determined by performing the molybdate test. This assay is based on the reduction of Mo(VI) to Mo(V) by fig syrup and subsequent formation of a green phosphate/Mo(V) complex at acid pH. The high absorbance values indicated that the sample possessed significant antioxidant activity. According to the results, fig syrup shows a significant antioxidant activity.

Furthermore, the ability of our sample to inhibit lipid peroxidation was evaluated by performing β-carotene-linoleic acid assay. In this model system, β-carotene undergoes rapid discoloration in the absence of an antioxidant, which results in a reduction in absorbance of the test solution with reaction time. This is due to the oxidation of linoleic acid that generates free radicals (lipid hydroperoxides, conjugated dienes and volatile byproducts) that attack the highly unsaturated β-carotene molecules in an effort to reacquire a hydrogen atom. When this reaction occurs, the β-carotene molecule loses its conjugation and, as a consequence, the characteristic orange color disappears. The presence of antioxidant avoids the destruction of the β-carotene conjugate system and the orange color is maintained [21]. Also in this experimental condition, good results were recorded, confirming the high antioxidant efficacy of the fig syrup.

Finally, the anticholinesterase activity of the syrup was evaluated. AChE plays an important role in the central nervous system. It is one of the fastest known enzymes and catalyzes the cleavage of acetylcholine in the synaptic cleft after depolarization. Inhibitors of AChE, such as galanthamine, are used frequently in the pharmacotherapy of Alzheimer Disease: AChE is, indeed, dramatically down-regulated in the brains of patients suffering from this disease [35]. Since a large amount of evidence demonstrates that oxidative stress is intimately involved in age-related neurodegenerative diseases, there have been a great number of studies which have examined the positive benefits of antioxidants to reduce or to block neuronal death occurring in the pathophysiology of these disorders [36].

The further good results obtained from cholinesterase inhibitory profiles demonstrated the real applicability of the syrup as a food supplement. 

## 5. Conclusions

In this work, a deep characterization of fig syrup, a Calabrian food product, and the efficacy of this fruit derivative as a nutraceutical supplement, was demonstrated by evaluating its antioxidant properties. Firstly, the content of antioxidant molecules, such as phenolic compounds and flavonoids, were determined by HPLC-DAD-MS analyses, Folin-Ciocalteu and AlCl_3_ methods. The high recorded values (3.92 mg of gallic acid equivalent per g of syrup and 0.35 mg of catechin equivalent per g of syrup, respectively), make this food product very interesting as an antioxidant nutraceutical. The determination of the total antioxidant activity confirms the high content of antioxidant equivalents in the syrup. The scavenger activities were investigated by performing DPPH and β-carotene-linoleic acid assays, showing the efficacy of the syrup in preventing damage induced by free radicals. Finally, the inhibition of the cholinesterase ability confirms the high benefits of this fruit derivative in human nutrition.
